# Supplementary material for: Frugal engineering of a jaw crusher using the factor-of-frugality, a modern version of the safety-factor
Source: PLoS One. 2025 Jun 24;20(6):e0325503. doi: 10.1371/journal.pone.0325503 (PMC12186939; doi:10.1371/journal.pone.0325503)
Supplement: Attachment — Submitted filename: Response to Reviewers.docx [file pone.0325503.s002.docx]

**Frugal engineering of a jaw crusher using the factor-of-frugality, a modern version of the safety factor**

At the outset we would like to thank the reviewers for their positive and valuable comments regarding this research effort. We also thank them for taking the time to point out meticulously issues pertaining to the content of this work. We have accordingly answered the various queries and revised the initial manuscript. Please find answers to the questions raised by the reviewers in the pages to follow. These useful comments have been used to revise the initial draft of our manuscript, which will be uploaded alongside these comments. Please note that the edited portions of the manuscript appear in red font in the revised manuscript (and which are underlined here). Also, some edits such as capitalizing certain words in the manuscript (shown in red-colored font) are not documented here for clarity and brevity.

**REVIEWER 1**

**Comment 1**: The title “Frugal engineering of a jaw crusher for extractive industries” is too general. It is necessary to specify the unique aspect of the study, for example, mention “factor of frugality” or “bio-inspired design”.

**Response:** The authors thank the reviewer for this suggestion.

**Action Taken:** *Page 1, Line 1-2:* Frugal engineering of a jaw crusher using the factor-of-frugality, a modern version of the safety factor

**Comment 2**: Similarly, it is worth adding more specialized keywords.

**Response:** The authors thank the reviewer for this suggestion and keyword listing has been changed.

**Action Taken:** *Page 1, Line 22-23:* *Jaw crusher, Factor of frugality, Sustainability, Frugal engineering, Frugal design, Frugal manufacturing, Biomimetics, Advanced frugal product*

**Comment 3**: The abstract gives a good overview of the study, but could be improved by adding quantitative results (for example, percentage weight reduction or increase in safety factor) for greater credibility. It is important to immediately indicate how much more “economical” the new design is in numerical terms.

**Response:** The authors thank the reviewer for this suggestion. And we have also added some minor content for a better reading of the abstract.

**Action Taken:** Page 1, Lines 15-19: Numbers indicated in the discussion and conclusion sections have been added in the abstract, as below.

Accordingly, *factors of frugality* of *1.87^1.79^*, *1.76^1.68^* and *1.63^1.33^* have been obtained for the flywheel, the Pitman and the rear wall, respectively, based on the frugal approach. Therefore, use of the new *frugal design approach* has resulted in material savings of 8%, 7% and 30% and increases in *factor of safety* of 35%, 71% and 18% in the flywheel, the Pitman and the rear wall, respectively, over their base values.

**Minor content:** Page 1, Lines 5-16:

Businesses are increasingly keen on going frugal due to increasing demand for sustainable and low-cost products that do not sacrifice quality. However, there is a dearth of tools for the systematic design and engineering of frugal products from scratch in the industry. Accordingly, a new approach has been applied in this paper for the formal design of a frugal jaw crusher for the mining industry. Consequently, this paper uses the *factor of frugality* (F of F^N^) which is a composite number that combines the *safety factor* (N) with fractions of material saved in various stages of product-development. In doing so, this work has iteratively applied the *factor of frugality* to the relevant components of a jaw crusher. And rigorous design procedures are adopted, for maintaining quality, due to the use of lower *safety factors* in making the product frugal. Contemporary concepts like generative design, *design for manufacturing* and biomimetics have been explored to achieve frugality in the relevant “bulky” components of a jaw crusher.

**Comment 4**: The introduction well justifies the relevance of frugal engineering, but lacks a clearer statement of the problem that this article solves. What is the specific “disease” of current solutions that the authors want to “cure”? Also, it would be worthwhile to expand the literature review on frugal engineering in the mining industry to demonstrate the novelty of the approach.

**Response:** The authors thank the reviewer for this suggestion. However, the authors would like to point out the following real-world problems that have been addressed in the paper:

1. Instead of quantifying the ‘frugality’ of a product purely using cost, a factor-of-frugality based approach is used, which captures the characteristics of going frugal, i.e., low-resource-consumption and better quality in addition to low-cost.
2. The focus of frugalization involving cost-reduction has also been shifted from the traditional avenues of labor and supply chain. Instead, frugal engineering principles have been applied at the design stage to directly influence material and cost savings while maintaining top quality. The application at design stage significantly helps in involving frugality in all stages of product development.
3. Up until now, the factor of frugality formula was developed to frugally engineer a single component of a system. A method to quantify the frugality of an entire assembly has been introduced in this paper.

In the introduction, it is also noted that this paper presents the first-ever instance of applying the factor-of-frugality based approach to an industrial product. Literature review of frugal products in the mining industry did not yield any results as products in the mining industry are typically characterized by bulky designs where robustness is more important than cost. Hence the absence of literature on frugal design from mining area which does not exist. In the interest of material savings and sustainability, a novel frugal design approach is adopted.

**Action Taken:** Accordingly, the issues that the paper addresses have been explicitly mentioned in the introduction with inclusion of previous content under point number 4.

*Page 3, line 76-91:*

Therefore, this paper addresses the novelty of applying frugal engineering principles to a jaw crusher by focusing on:

1. Frugal design is quantified using the *factor of frugality*. This allows the quantification of frugality in any product, including the jaw crusher, using a metric that hews with the three features of frugal engineering, i.e, low-cost, low-resource, and better quality.
2. The impact of the *factor of frugality* based design has been studied, using performance and cost as metrics.
3. The *factor of frugality* has been formulated for design of individual components of a given product. Consequently, a method of determining the overall *factor of frugality* of an assembly based on this formulation has been introduced in this effort.
4. This paper is the first instance of the methodical application of the *factor of frugality* to an actual industrial product. Accordingly, this paper presents the different versions of the product resulting from the iterative application of the *factor of frugality* while converging onto the final design. The advantages and limitations of the frugal methodology will become apparent in the process of designing this industrially relevant product.

**Comment 5**: “Use of the factor of frugality” – the very concept of “factor of frugality” requires a more detailed explanation. How is it calculated, what parameters are taken into account, why these particular ones? In formula (1), it is necessary to explain what “i” (index) is and what specific “material savings schemes” are implied.

**Response:** We thank the reviewer for this comment. Since this is a new factor with a new concept, we have provided details on pages 5-8 and Figures 2 &3. We have added new content to make this clear. We feel that the existing and added content on this new factor makes this concept clear. But the interested reader can look, if needed, for more details into Rao’s works that are referenced in the paper.

**Action Taken:** Accordingly, the following lines have been added.

*Page 5, Lines 120-123* : The factor of frugality is a new metric for design and, engineering in general, that achieves best functionality under resource-and-cost constraints by subsuming the classical safety factor while also focusing on individual stages in product development. It is a modern version of the factor of safety that focuses on safety, resource, cost and quality in design.

*Page 5, Lines 124-126:* The *factor of frugality* approach is initiated by fixing N at a low value of 1.5, as seen in Fig 2 [3]. The low value of N is arbitrary in that it should be lowest possible value commensurate with the current body of knowledge in a given area of engineering.

*Page 6, Lines 129-138:* Fig 2 brings out the working of the *factor of* frugality, i.e, F of F^N^, for a shaft which is a workhorse of many engineering applications. As seen in Fig 2, a low value of N, at 1.5, is fixed throughout the *frugal product development* process. Such a low value of N generally results in lower material consumption and subsequent stages of *product development* are examined for more material savings in addition to that for this low value of N. Consequently, the *material saved* (MS) parameters account for weight of material saved in the design, materials and manufacturing stages with focus on salvaging or recycling. Therefore, low-resource consumption and, hence lower costs, are made possible from both low N and individual stages of *product development.* The quality is maintained at the highest level by hewing to the rigor of most accurate design and engineering principles.

*Page 6, Line 141:* However, some changes with this idealized setting of the *factor of frugality* are in order.

**Comment 6**: The numerical modeling section lacks information on model validation. How is the adequacy of the finite element analysis model used confirmed? References to relevant standards, experimental data, or comparison with other known models are needed.

**Response:** Page 9, Table 2 furnishes details about the type of mesh used and convergence criteria used. The h-refinement of the mesh is stopped once max stress deviation is within 5% of that of the previous iteration. This is done so that a mesh independent result is achieved. This gives sufficient confidence for the convergence of the result. Furthermore, all analyses are done within the Hookean domain, with none of the analyses exceeding the yield strength of the material. This has been specified in the paper.

We understand the reviewer’s concern on model validation. This is the first part of a study that aims to build and use such a *frugal* jaw crusher. We hope to publish these results when they become available.

**Action Taken:** Some minor modification was carried out.

*Page 8, Line 182:* …domain since plastic flow is detrimental…

**Comment 7**: Version 1 (the basic design) is described as “simple”, but there is no detailed explanation of why this particular design was chosen as a starting point. Version 2 is created using the generative design method. However, there are few details about the parameters and constraints used in the generative design. Version 3 is bio-inspired, but the connection to specific natural objects is not obvious.

**Response:** We thank the reviewer for this comment. The base model (Version 1) is a concept that is made in CAD based on machines observed in the field. This base design is chosen as a starting point because these designs have a low factor of safety, less than or equal to 1.5, as recommended by the frugal design methodology specified in Rao’s works. Subsequent design iterations aim to reduce material without decreasing this factor of safety. In Version 2 of the flywheel, which employs generative design, it is mentioned that symmetry constraints were applied on mutually perpendicular planes passing through the axis of the flywheel (Page 11, line 249-250). This results in a symmetric design. In all three versions, a torque and a belt tension force are also applied as loads on the rim of the flywheel (Page 10, line 225). These are the constraints and boundary constraints that are required to simulate loads on a flywheel. As for the connection to biomimetics, the connection to trees and branching etc have been briefly explained and the interested reader can refer [41,42] under Version 3 on page 12. We believe that relevant biomimetic features have been briefly explained here.

**Comment 8**: Tables 3, 4, and 5 present the results for each version of the components. However, there is no detailed analysis of these results. Why do some parameters improve while others deteriorate? What are the reasons for these changes? It is necessary to relate the results to the specific design features of each version.

**Response:** We thank the reviewer for this comment. Tables 3, 4 and 5 are meant to be observations. An analysis of these results can be found in the ‘Discussion’ section. The changes between each subsequent iteration of the design is necessitated by the frugal design methodology shown in Figure 3. The details of these changes with reference to the numbers shown in the tables can be found in the ‘Results’ section, refer Page 9 – 17. However, we have added two paras to account for this comment as follows.

**Action Taken:** *Page 19 -20, lines 397 – 420*:

A brief analysis on how iterative changes in design affected the F value of each component now follows. As shown in Fig. 12, the F value of the flywheel increased from *1.32* to *1.72* in the first iteration. This is attributed to the application of generative-design and DFM principles. In particular, engineering the arms of the flywheel to better handle bending reduced the stress concentration, thereby enabling material reduction through stronger design. Further refinement in the second iteration was achieved by following again, DFM rules specific to flywheel-manufacturing and, by using principles of biomimetics for mimicking the structure of trees. The details of these design changes have been covered under the section on results. Consequently, additional branching and tapered profile of the flywheel-arms resulted in a stronger structure with lesser material, which has been captured in the final F value of *1.88*, see Fig. 12.

Similarly, the first iteration of design for the Pitman frame, shown in Fig. 12, increased the F value from *0.98* to *1.54*. This can be attributed to the change in design of the lower half of casting, wherein a webbed rib was adopted with larger fillets. This allowed saving on weight while improving strength. The second iteration of the Pitman sees an increase in F value from *1.54* to *1.76*. The focus in this iteration was on the structure of the frame itself. A twin-wall structure with a beaded rib was adopted for design. As outlined in the section on results, this design leverages the combined advantages of biomimetics and traditional engineering concepts. As for the rear wall, the casting went through a single iteration where the F value increased from *1.13* to *1.63*, see Fig. 12. The design change involved removal of material in regions of low stress where the material was redundant. Such material reduction combined with larger fillets resulted in a lower peak stress and uniform stress distribution, as shown in Fig. 10 and Table 5. The combination of material savings and stronger design contributed to the significant uptick in F-value of the rear wall.

**Comment 9**: It is important to show that the “factor of frugality” is not just an abstract value, but actually reflects resource savings and increased safety. What is the correlation between the “factor of frugality” and other performance indicators (e.g. production cost, service life, reliability)?

**Response:** We thank the reviewer for this comment. We have explained these features in bringing out the concept underlying the Factor of Frugality on pages 5 to 8. We have also added new content to explain the theoretical underpinnings of this new design-metric that captures both the resource savings and safety of a frugally engineered product while keenly focusing on cost and top quality. This has also been explained in detail in Rao’s works [2,3,12,18,19, 28].

As for the application to an industry grade jaw crusher, an increase in *factor of safety* from design iterations would no doubt improve reliability and service life, which are both functions of the *factor of safety*. A more detailed investigation into such indicators is currently not within the scope of this effort. However, our group is investigating topics like optimum values of the *safety factor* and *reliability* in various sectors and their impacts on product development, costs etc. However, our initial foray into jaw crushers has shown that the production costs are lowered due to the commensurate material savings mentioned in the paper. This is shown by the cost savings workup given in the initial draft of our paper. Please refer to Table 6 on Page 22.

**Comment 10**: The presented cost savings calculation is too simplified. It does not take into account the costs of developing new designs, making prototypes, conducting tests, and possible changes in the technological process. Formulas (4) and (5) use coefficients 1.1 and 1.3 to account for metal losses and savings on sprues. It is necessary to justify the choice of these coefficients with references to literature or your own calculations.

**Response:** We thank the reviewer for this comment. It must be noted that the cost of a product has two components to it: fixed and variable. The reviewer’s comments pertain to the ‘fixed’ component of the costs, which is going to remain constant through the different design iterations as there is no change in the process, as noted in the paper. Hence, the variable cost component of the component is considered, which has a direct correlation to material savings.

The choice of these coefficients in formulas (4) and (5) is justified by the reference cited [45] and the values are well within the quoted range. These are standard values assumed during cost estimation, based on typical values for castings. Also, these are cost ‘estimates’ and not actual costs as costing can be highly subjective based on various factors.

However, the reviewer is right is pointing out the simple nature of these initial cost analysis. We do hope to carry out detailed costing to strengthen our initial analysis which has shown the potential of low-cost in going advanced-frugal. We have attempted to present all the features such as low-resource and functionality in addition to low-cost for achieving such an advanced-frugal-product. And, each of these features can be expanded upon which we hope to accomplish in the foreseeable future.

**Comment 11**: All indicators are highly dependent on the material. It would be good to consider other materials and compare the indicators.

**Response:** We thank the reviewer for this comment. We are aware that the indicators are material-dependent. To this effect, the Factor of Frugality also accommodates material changes in its calculations, please refer Figure 3. However, the objective of this paper is to compare the various design iterations of the product. If a material change is also introduced, an apple-to-apple comparison of the design alternatives would be impossible. The amount of material in the product would also vary depending on the design changes induced by the material change. Furthermore, it would also entail a lengthy cost-benefit analysis that is beyond the scope of this paper. Hence, material changes have not been considered within this paper to focus on frugalizing the design. We hope to include such a study in our future works.

**REVIEWER 2**

**Comment 1**: Although this paper represents a very interesting topic to the field and probably considered all processes, however it relies on granite as the crushed material while in nature the granite as raw material may be found mixed with other material particles such as iron. It’s recommended to take that into consideration.

**Response:** We thank the reviewer for this comment. We are aware that granite itself is a mineral that varies in property depending on where the sample is taken from and what weaknesses are present in the bulk of the material. The objective of this work was to establish a base design and load conditions based on the references cited. Calculations that consider variations in the properties of granite itself would be out of the scope of this paper. However, it is worth noting that even if a harder granite is fed into the jaw crusher, the jaw crusher would still accept a slightly smaller feed size. Aside from this minor difference, we anticipate no major impact due to the changes in the properties of the feed material.

**OTHER CHANGED CONTENT**

**Introduction:**

We have added an extra reference number 28 in some places in the revised manuscript.

*Page 3, Lines 60-61:* frugal product, the exclusion of resource-consumption and quality [17] would significantly hinder creation of a truly advanced frugal product.

*Page 3, Line 75:* uncertainties arising from lower *safety factors* [3,18,19].

*Page 4, Line 94:* **Selection of a Suitable Industry Level Product**

*Page 4, Line 102*: ….relevant “bulky” components from this product using…..

*Page 4, Line 104:* …. and impact between a fixed- and a moving-jaw.

*Page 5, Line 124:* The *factor of frugality* approach is initiated by fixing N at….

*Page 7, Line 148:* ….typically be an existing “bulky” design…

*Page 7, Line 151:* …is F^N^ where F is…

*Page 8, Line 170:* …incorporates modern design concepts like biomimetics and….

**Results:**

*Page 9, Lines 191-192:* The crusher is designed for a power of *110 kW* at a shaft-speed of *230 RPM* with a feed acceptance of *600 mm* [20-26]. The material to be crushed is granite.

*Page 9, Lines 195-196:* due to the corrugated nature of the liner plates.

*Page 9, Line 201:* where,

*Page 9, Lines 202-205:* “is” has been inserted

*Page 10, Line 206:* For *L = b = d = 0.6 m* and a σ of *25 MPa…*

*Page 10, Line 210-212:* The toggle plate is designed to fail at a force of *1.5 × 10^6^ N*, which according to Eqn (2), corresponds to a cubic rock of side 0.3 m being broken at the lower portion of the crusher, assuming the rock has fallen to about 75% of the length of the pitman liner.

*Page 12, Line 251:* A rounded rectangular cross section with…

*Page 12, Line 265:* The odd number of spokes and the curved design, as per recommendation in [31],…

*Page 12, Line 268: …*designed four-spoke structure, distributing…

*Page 13, Line 282:* The pitman frame performs the vital function of…

Page 14, Line 303: Fig 8. Stress analysis of the Pitman frame - Rear view shown in insets

*Page 15, Line 314:* Table 4. Summary of results for the Pitman frame design

*Page 15, Line 316:* Version 3, shown in Fig 7, is a biomimetics-design…

*Page 15, Lines 319-322: …*close to 2 for animals that had the strongest bite force per unit length of their jaw. Also, this version of the Pitman frame has a ratio of 2.1 between the length up to the toggle seat and total height of the Pitman frame**,** which is ideal as per the observations on the measurements of animal jaws.

*Page 15, Line 325:* Although this design resulted in a slight….

**Discussion:**

*Page 18, Lines 382-384:* The theoretical model for the F^N^ approach fixes the value of N at 1.5. However, when dealing with complex structures, occurring in real time, such as those in this…

*Page 18, Line 393:* …values of each design version is shown in Fig 12.

*Page 20, Line 422:* … and MS. This figure quantifies different…

*Page 20, Line 426: …* extraneous *material saving* schemes…

*Page 21, Line 430: …* adoption of biomimetics-based design and…

*Page 21, Lines 439 - 443:* Accordingly, cost-benefit accrues from the use of lesser material and reduced expenditures associated with the casting process. As for the latter, a lighter casting leads to proportional savings through reduction of wastage. This is because the weight of runners and risers, i.e., gating system necessary for molding a casting, is proportional to the weight of the casting itself.

*Page 22, Line 471:* The *frugal design approach* was used in improving the design of…

*Page 22, Line 476:* Accordingly, if the toggle…

*Page 22, Lines 478-479:* This is based on using granite with a modulus of rupture of *25 MPa*.

*Page 23, Lines 485-488:* Although testing is required to validate these numbers, the design methodology, based on the factor of frugality, shows the ability to improve performance while also saving on material use. Therefore, the potential of this framework for the systematic design of advanced frugal products, such as the jaw crusher, has been demonstrated in this paper.

*Page 23, Line 509:* Therefore, the composite F_ASSEMBLY_ for…

*Page 23, Lines 510-512*: A value of *1.5* will be assigned to the remaining components listed in Table 1, as per the initial value recommended for the frugal design process [3]. This is assuming that these remaining components satisfy the basic functional...

Pages 23-24, Lines 514-516: Accordingly, F_ASSEMBLY_ is determined, from Equation (6) and Table 7, to be *1.74* for the final, frugally engineered jaw crusher. In contrast, the F_ASSEMBLY_ of the initial design of the jaw crusher is lower at *1.37.* Therefore, for *~27%* increase in F_ASSEMBLY_, the jaw crusher...

*Page 24, Table 7:* Added columns ‘F_initial_’ and ‘M_1_* F_initial_’. Also ‘M_1_*F’ modified to ‘M_1_*F_final_’

**Conclusions:**

*Page 24, Lines 527-532:* The design approach based on the *factor of frugality* yielded material savings in the range of *7% - 30%.* In all cases, material savings have been achieved between the iterations. It also increased the *factor of safety* by at least *18%* in the components studied. The *factor of safety* in the final iteration is slightly higher than the targeted value of *1.5*, thereby leaving room for further material savings through design optimization.

*Page 24, Lines 536-539:* The *factor of frugality*-based design-approach yields significant material savings. This translates to significant cost savings, including expenditures from attendant energy consumption. The case for lower costs is further strengthened when components are frugally manufactured in emerging markets like India.

*Page 25, Lines 540-544:* The *factor of frugality* goes beyond the *classical factor of safety* in looking for more material savings while maintaining performance-quality at low-cost. Therefore, the *factor of frugality* is a modern version of the traditional *factor of safety* that will help designers engineer their parts/products for low-cost and low-resource in addition to safety through best performance.
